# Supplementary material for: Electrical injection and detection of spin-polarized currents in topological insulator Bi2Te2Se
Source: Sci Rep. 2015 Sep 22;5:14293. doi: 10.1038/srep14293 (PMC4585757; doi:10.1038/srep14293)
Supplement: Supplementary Information [file srep14293-s1.pdf]

Supplemental Materials

Electrical injection and detection of spin-polarized currents in topological insulator Bi_2Te_2Se

Jifa Tian[1,2,*], Ireneusz Miotkowski[1], Seokmin Hong[2,3], and Yong P. Chen[1,2,3,*]

1. Department of Physics and Astronomy, Purdue University, West Lafayette, IN 47907, USA

2. Birck Nanotechnology Center, Purdue University, West Lafayette, IN 47907, USA

3. School of Electrical and Computer Engineering, Purdue University, West Lafayette, IN 47907, USA

*Email: tian5@purdue.edu; yongchen@purdue.edu

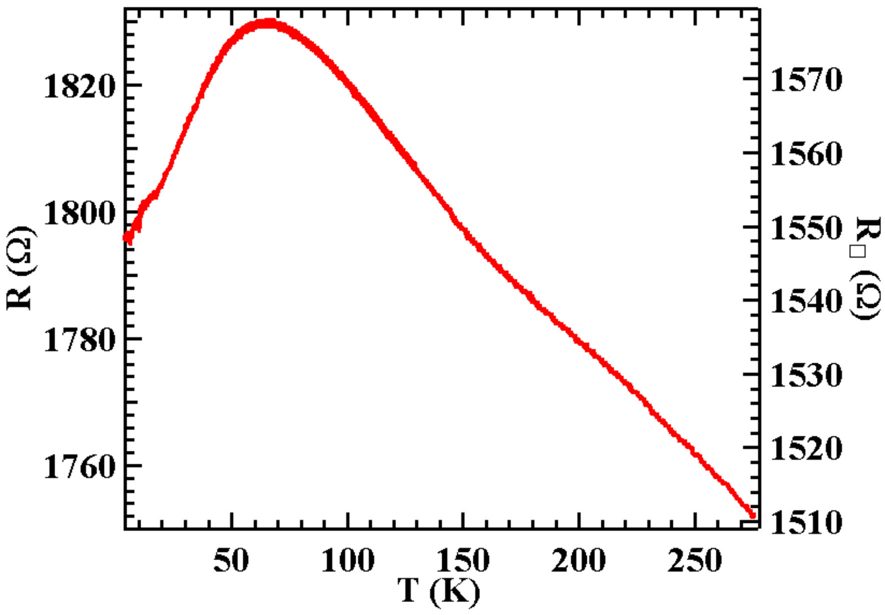

Figure S1 Temperature dependence of the resistance of an exfoliated 39 nm-thick BTS221 flake. Right axis shows corresponding sheet resistance. Note such reference devices have only Au contacts and did not go through as many fabrication steps compared to the spin devices in the main text.

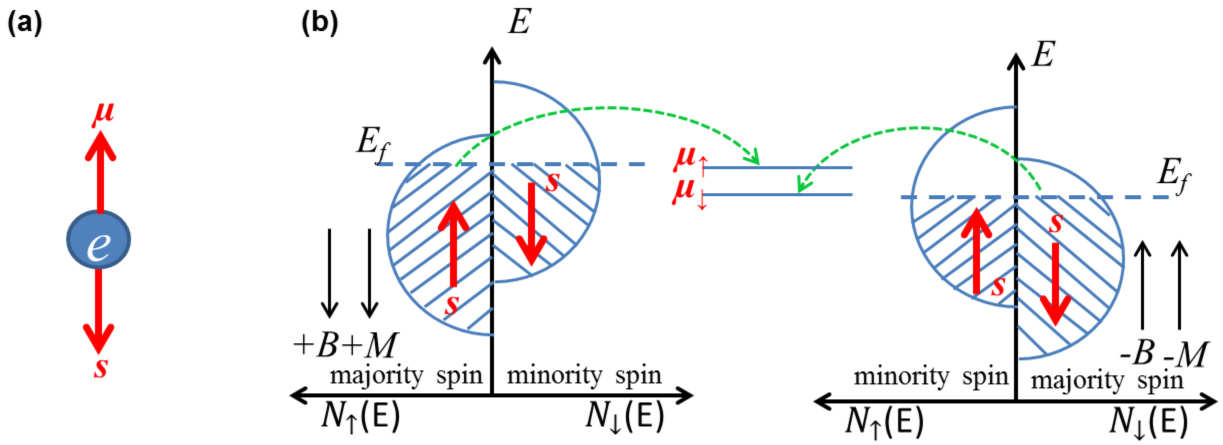

Figure S2 Schematics of magnetization and spin polarization of ferromagnet (FM) and how it measures a spin polarized channel. (a) Schematic representation of the relative orientation of magnetic moment and spin of an electron. The magnetic moment $\boldsymbol{\mu}$ is opposite to its spin $\boldsymbol{s}$ (the direction of the spin angular momentum) due to the negative charge of electron [S1-S3]. (b) Schematic density of states (DOS) diagrams of a ferromagnetic with +$\boldsymbol{M}$ (shown on the left) and –$\boldsymbol{M}$ (right), respectively. The orientation of the majority spin (which determines the magnetization) is opposite to the magnetization direction. A channel with finite “up” spin polarization is depicted in the middle (with more occupation, or higher chemical potential of the “up” spins). When used as a detector (voltage probe) in spin transport (spin potentiometric) measurements, the FM will mainly connect with (and measure the corresponding chemical potential of) the channel spins whose orientation is parallel with that of the FM majority spins [S3-S7]. For example, the FM with down (up) magnetization +M (or -M), or equivalently up (down) majority spin orientation, will mainly measure the up (down) spin electrochemical potential in the channel (depicted by the dashed connections).

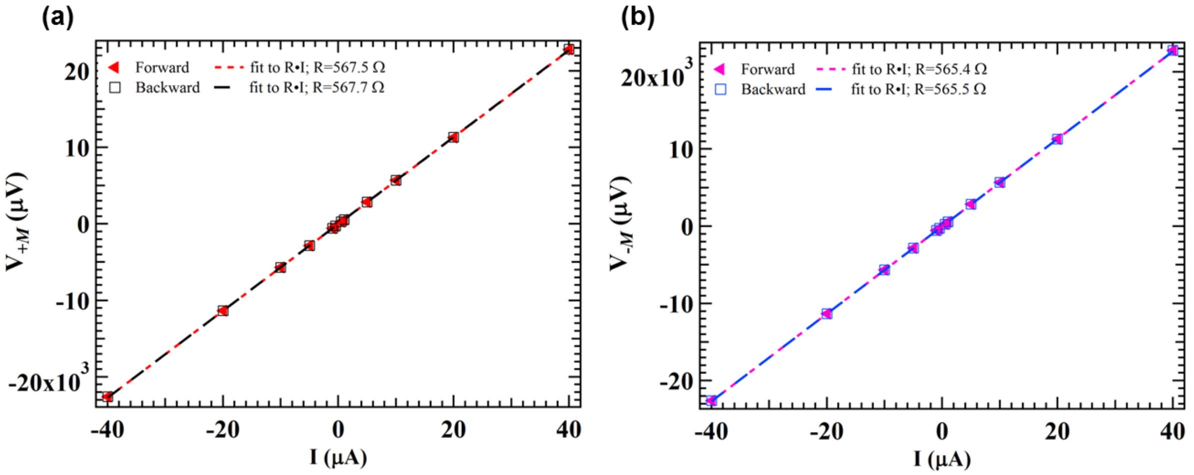

Figure S3 (a) $V_{+\boldsymbol{M}}$ and (b) $V_{-\boldsymbol{M}}$ measured by the Py spin detector on device *A* as a function of the bias current *I* for both forward and backward magnetic field sweeps, respectively. Here, $V_{+\boldsymbol{M}}$ and $V_{-\boldsymbol{M}}$ are the

average voltages measured between (0.04T, 0.06T) and (-0.06T, -0.04T) as shown in Fig. 3, respectively. The dashed lines in (a) and (b) are the corresponding linear fittings to R×I. We extract R to be 567.5 Ω (forward) or 567.7 Ω (backward) for $V_{+\boldsymbol{M}}$, and 565.4 Ω (forward) or 565.5 Ω (backward) for $V_{-\boldsymbol{M}}$. The difference between R for +***M*** and –***M*** is δR~2 Ω. Due to the very thin Al_2O_3 used, the IV can still be largely ohmic.

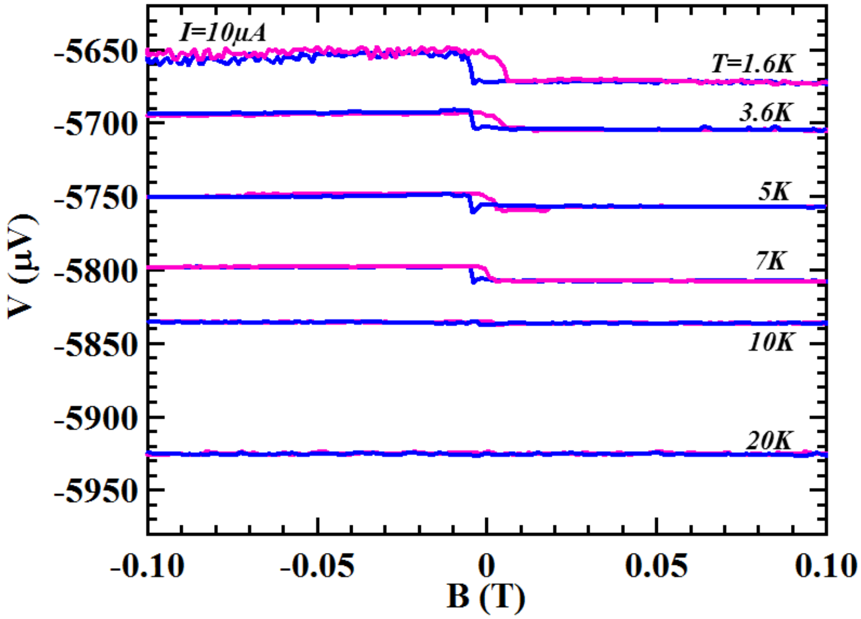

Figure S4 Voltage measured by the Py spin detector on device *A* as a function of in-plane magnetic field under a DC bias current of -10 μA at the same set of temperatures shown in Fig. 4a (from 1.6 to 20K). The five higher-*T* (from 3.6K to 20K) curves are vertically offset by consecutive integer multiples of -35 μV for clarity.

References:

S1 C. Kittel, "Introduction to solid state physics". 8th ed., Wiley, (2004).

S2 B. T. Jonker, A. T. Hanbickia, D. T. Pierceb, M. D. Stiles, "Spin nomenclature for semiconductors and magnetic metals", J. Mag. Mag. Mater. 2004, 277, 24–28

S3 Handbook of Spin Transport and Magnetism, edited by Tsymbal E. Y.; Zutic, I. CRC Press 2011.

S4 R. Meservey and.P. M. Tedrow, "Spin-polarized electron tunneling", Phys. Rep. 1994, 238, 173-243.

S5 P. R. Hammar and M. Johnson, "Potentiometric measurements of the spin-split subbands in a two-dimensional electron gas", Phys. Rev. B 2000, 61 7207.

S6 M. Johnson, "spin injection and detection in a ferromagnetic metal/2DEG structure", Physica E 2001, 10, 472-477

S7 Y. H. Park, H. C. Jang, H. C. Koo, H.-J. Kim, J. Chang, S. H. Han and H.-J. Choi, "Observation of gate-controlled spin-orbit interaction using a ferromagnetic detector", J. Appl. Phys. 2012, 111, 07C317.
